# Confined space airway management: a narrative review

**DOI:** 10.1186/s13049-025-01357-8

**Published:** 2025-05-05

**Authors:** Soren S. Rudolph, Christopher W. Root, Michael Friis Tvede, Trond Fedog, Patrick Wenger, Mikael Gellerfors, Jelsche Apel, Luca Ünlü

**Affiliations:** 1 Department of Anaesthesia and Trauma Center, Centre of Head and Orthopaedics 6011, Copenhagen University Hospital Rigshospitalet, Copenhagen, Denmark; 2 The Danish Air Ambulance, Brendstrupgårdsvej 7, 8200 Aarhus N, Denmark; 3 Department of Emergency Medicine, University of New Mexico Health Sciences Center, Albuquerque, NM USA; 4 Air Zermatt, Emergency Medical Service, Heliport Zermatt, 3920 Zermatt, Valais Switzerland; 5 Swedish Air Ambulance (SLA), Mora, Sweden; 6 Ambulance Care in Greater Stockholm LTD, HEMS, Stockholm, Sweden; 7 Department of Perioperative Medicine and Intensive Care, Karolinska University Hospital, Stockholm, Sweden; 8 Department of Anaesthesiology, Intensive Care and Emergency Medicine, Cantonal Hospital of Graubuenden, Chur, Graubuenden Switzerland; 9 Swiss Air-Ambulance, Rega, PO Box 1414, 8058 Zurich, Switzerland; 10 Department of Emergency Medicine, University Hospital Basel, Petersgraben 2, 4031 Basel, Basel Switzerland; 11 Faculty of Medicine, University of Basel, Klingelbergstrasse 61, 4056 Basel, Basel Switzerland

**Keywords:** Airway management, Emergency medical services, Tracheal intubation, Supraglottic airway devices, Confined spaces, Prehospital care

## Abstract

**Background:**

Airway management is a critical component of prehospital and emergency care, often complicated by the environment in which it is performed. Confined space airway management (CSAM) refers to scenarios were restricted physical space challenges conventional airway techniques. These situations may occur in unpredictable environments, such as vehicle entrapments or collapsed structures, and controlled settings like helicopters. This narrative review aims to synthesize current knowledge, expert opinions, and evidence on CSAM.

**Main body:**

CSAM poses logistical and technical challenges, including limited access to the patient, restricted movement, and reduced visibility. These factors increase the difficulty of performing standard airway management procedures and increase the risk of complications. Supraglottic airways (SGA), due to their ease of insertion and high success rates, are recommended as a first-line approach in CSAM, especially when intubation is delayed or infeasible. Tracheal intubation (TI) may require significant modifications in technique. Alternative methods and adjuncts such as face-to-face intubation and stylets may be considered but are highly dependent on provider expertise and the specific scenario. Emergency front of neck access (eFONA) is provided with high success rated in confined spaces. In controlled settings, systematic preparation can improve success rates and reduce procedural times. In uncontrolled environments, prioritizing patient extrication and maintaining oxygenation is essential, as definitive airway management may conflict with rescue efforts.

**Conclusion:**

CSAM requires a strategic blend of medical expertise, adaptive techniques, and logistical planning. A focus on training, preparedness, and the use of supraglottic airway devices may mitigate challenges in these high-stakes scenarios.

## Introduction

Airway management is critical to emergency medicine, anesthesia, and critical care. In controlled settings like operating rooms with ample space, advanced monitoring tools, and a team of experienced professionals operating under standardized protocols, airway procedures are performed with high success rates and minimal complications [1].

Complication rates rise substantially when airway management is performed outside the operating room, particularly in unscheduled emergencies, during odd hours, under variable environmental conditions, and in critically ill or injured patients—all characteristic challenges of prehospital airway management [2, 3]. The cornerstone of successful prehospital airway management is optimizing the environment to facilitate optimal patient access and implementing a standardized approach that mirrors in-hospital procedures [2–14].

However, environmental constraints often cannot be avoided, and airway management must proceed without delay. In some cases, the environment may be familiar and conducive to established protocols, such as in a helicopter cabin or the back of an ambulance. In others, the environment may be unpredictable or restrictive, severely limiting the movement of both the patient and the emergency medical provider. These scenarios known as Confined Space Airway Management (CSAM), introduce unique challenges and complexities that require specialized skills, tailored equipment, and adaptations of standard airway management procedures [10, 15–19].

This narrative review is a collaborative effort by an international group of physicians and paramedics specializing in anesthesiology, emergency medicine, intensive care, and prehospital medicine, who also serve as faculty for The Big Sick Conference airway workshop. It aims to synthesize existing literature and expert opinions on confined space airway management (CSAM).

## Methods

A non-systematic search was conducted in PubMed, Cochrane Library, CINAHL, and Embase in April 2024. To allow for a comprehensive understanding of the different approaches to managing airways in confined spaces the search included all study designs, all patient populations. No restrictions were applied regarding language, publication date, or geographical origin of the publications. Two authors (SSR, MFT) independently reviewed the search results and identified relevant publications for inclusion in this narrative review. Discrepancies were resolved through discussion.

An abstraction sheet was developed to systematically record key details of the studies, including the year of publication, country, population, number of patients/participants, methods, interventions, outcomes, results, and conclusions. Each study was independently reviewed and abstracted by two authors to ensure objective and consistent data extraction. Additionally, the abstractors cross-referenced the reference list of each full article to identify relevant publications not captured in the initial search (e.g. society statements not indexed in electronic databases). Any disagreements between the abstractors were resolved through consultation with one of the senior authors (SSR, CWR).

## Discussion

### Confined space–defining controlled and uncontrolled

A confined space is defined as an enclosed or partially enclosed environment with restricted access to the patient, which can complicate the delivery of medical interventions and hence airway management.

Confined space scenarios typically occur in **uncontrolled** environments where opportunities for specific task planning and preparation are limited. Entrapped patients are for example encountered in vehicles with significant structural deformation following high-impact forces, as well as during rescue operations within collapsed buildings, industrial settings, heavy machinery, air ducts, crawl spaces, avalanches, or manholes.

Uncontrolled confined space or entrapment scenarios in the prehospital environment is of notable concern due to clinical risk for the patient and occupational risks for the rescuers [16, 20–22]. The incidence of these scenarios is probably underreported. A systematic review identified that up to 40% of patients will remain trapped in their vehicles after motor vehicle collision [23]. One multi-center prehospital study conducted in Germany in 2006 even reported that limited access to the patient, mainly because of entrapment, was found in 20.2% of the patients and this was present in 9.6% at the time of tracheal intubation (TI) attempt. [24]. Other studies have inferred that the prevalence of complications arising from entrapment during medical interventions are considerable [12].

Conversely, in **controlled** environments like the cabin of a helicopter, an airplane, or an ambulance the opportunity for planning, preparation and training allows for a more structured approach to airway management, reducing the unpredictability and complexity associated with confined spaces in uncontrolled settings [15, 17, 20]. Inside the hospital, a tightly packed modern operating theater may also generate confined spaces. However, challenges in these confined spaces are amenable to proper planning and training [12, 16, 20–22, 24, 25].

### Confined space airway management adjuncts & devices

Numerous studies have evaluated various adjuncts and devices for CSAM. Most of this research has been performed in simulated environments using manikins in confined spaces with restricted access or entrapment scenarios, while a smaller proportion consists of limited case series.

#### Dust masks

Airborne particles may cause dust impaction and progressive loss of airway and respiratory problems in spontaneously breathing patients. Position statements such as of the National Association of EMS Physicians (NAEMSP) and the Federal Emergency Management Agency advocate that patients in potentially dusty environments may benefit from early use of dust masks [18, 26].

#### Bag-valve-mask (BVM)

While Bag-Mask-Ventilation (BMV) is considered an essential skill, achieving effective ventilation is generally difficult in the resuscitative setting [27, 28]. Even more so in CSAM, where the patient’s position might not be optimal for BMV. Two simulation studies investigated the efficacy of BMV in entrapped manikins. In the first study from 2007, 8/38 (21%) participants succeeded with facemask ventilation from the side and 21/38 participants (55%) from the backseat. Gastric inflation was noted in all cases. In another manikin study, overall success rate for BMV was 97% [29–31].

#### Manual in-line stabilization (MILS)

Manual In-Line Stabilization (MILS) of the cervical spine is widely accepted and applied during CSAM. Balancing spinal protection and effective airway management is key. Use of SGAs require less cervical movement, making them useful alternatives to TI. Video laryngoscopy may improve visualization with minimal cervical movement.

Based on rescuer positioning, nature and size of the confined space and availability of additional personnel MILS may need to be adapted. In *Rescuer-Supported MILS* situations when a second rescuer is unavailable; the airway provider may use their hand, forearm, or knees to stabilize the patient’s head while intubating. In *Angled MILS*, the provider must intubate from the side or behind, they should maintain in-line stabilization using a partner. In *Reverse MILS*, the rescuer provides spinal stabilization from behind the patient’s head instead of stabilizing from the front. *Passive MILS* utilizes available debris or materials (sandbags, towels, foam pads) to stabilize the cervical spine instead [32, 33].

#### Supraglottic airways (SGA)

Supraglottic airways (SGA) play a crucial role in emergency airway management both as primary and backup plan [1, 32, 34]. In the context of CSAM, SGAs may be beneficial due to their ease of insertion and high success rates. Several SGAs have been studied including the Classic Laryngeal Mask Airway, LMA Supreme®, iGel®, Combitube®, Ambu King LTS Laryngeal tube® and the LMA Fasttrach®/Intubating LMA.

When compared to TI by both direct and video laryngoscopy, previously mentioned SGAs have shown comparable or higher success rates (65–100%) for insertion and shorter times to effective ventilation in various studies. [29, 30, 35–48].

The effectiveness of both SGA and TI depends on the training and experience of the healthcare provider. In the confined space setting, SGAs are particularly valuable because they do not require the same degree of space and access to the patient’s head that is required for BMV or laryngoscopy and TI. Furthermore, insertion of SGAs does not require the use of muscle relaxants. While SGAs may be easier to use for less experienced providers, TI may be preferred by seasoned clinicians who have extensive training in advanced airway management techniques [6, 12].

#### Tracheal intubation

Tracheal intubation (TI) typically requires more complex techniques and may necessitate additional equipment, making it less practical in tight environments. In confined spaces, TI poses a higher risk of complications, airway trauma, and multiple attempts. The success rate of TI may decrease significantly [37, 38].

When comparing TI using direct laryngoscopy (DL) versus video laryngoscopy (VL) of any type, there is a theoretically advantage to VL given that it allows for an indirect line of sight to the glottic opening which would seem important in the entrapped patient with limited access to the head. Despite a significantly improved view of airway structures, times needed for successful TI, and overall success rates have not been shown to be higher for VL [37, 49, 50].

Data does not support distinction between VL devices using standard geometry blades, channeled devices or hyper angulated blades concerning success rates in confined spaces. However, several studies argue for the use of a channeled devices such as the Pentax Airway scope®, the Airtraq® or Kingvision®, as they may be advantageous in situations where the rescuer’s position is very awkward relative to the patient’s head [35, 42, 49, 51–54]. In contrast, a well-conducted prehospital RCT showed that the Airtraq® had a significantly lower tracheal intubation (TI) success rate compared to the direct laryngoscope (47% vs. 99%), raising questions about its effectiveness for prehospital TI [55].

Tracheal tube introducers and stylets are essential adjuncts in in-hospital and prehospital airway management. Their use has been linked to higher first-pass success rates, especially when combined with video laryngoscopy [56]. A small manikin study simulating an entrapment scenario in a car assessed the Steerable Tracheal Intubation Guide (S.T.I.G.)—previously known as the Flexible Tip Bougie (Construct Medical Pty Ltd., Hawthorn, Australia)—during direct laryngoscopy. The study found that the S.T.I.G. was not superior to standard bougies in terms of first-pass success rates or intubation time [57]. However, the paramedics in this study were introduced to the steerable tip bougie just before participating.

Blind TI via the LMA Fasttrach®/Intubating LMA (ILMA) has shown a comparable success rate to DL in confined space manikin studies [35, 36, 43, 58]. The ILMA has largely been replaced by second-generation SGAs and VL and is no longer considered a primary airway device. Its role is diminishing in updated guidelines, but it may still be found in older protocols or settings where advanced devices are unavailable.

The use of an endotracheal tube equipped with a camera at the tip (AMBU VivaSight®), combined with direct laryngoscopy (DL), was evaluated in a simulated scenario involving entrapped patients in vehicles with restricted access. In this small manikin study, paramedics had significantly better first-pass success rates (45/45, 100%) compared to DL alone (39/45, 87%) when using the AMBU VivaSight® [59].

The Vie Scope® is an illuminated intubating device with a design similar to a straight Miller blade. The intubation technique using the Vie Scope requires the use of a bougie, which is inserted first and then after removing the scope the endotracheal tube is railroaded over the introducer. In a small manikin study, the Vie Scope® required significantly longer time for TI (35.03 s (± 5.27) vs. 51.1 s (± 6.28), *p* = 0.02) when compared to standard Macintosh laryngoscopy [60].

Prehospital TI requires a higher level of training and familiarity with advanced airway techniques and induction of anesthesia. However, any standard practice can be complicated by the challenges posed by limited access and visibility. In one study paramedics had higher success rates than experienced doctors using the same device, which may be attributable to familiarity with the work environment [37].

### Tracheal intubation techniques in confined spaces

#### Face-to-face technique

The face-to-face or inverse technique has been considered an option for CSAM. The technique requires the airway provider to position themselves in front of the patient, the laryngoscope is typically held with the right hand, with the handle directed towards the patient’s feet giving rise to the names "ice pick", or "tomahawk" technique.

Several studies have examined TI using these techniques both in simulated scenarios and retrospective case series highlighting the associated success rates in confined spaces [6, 12, 16, 36, 51, 53, 61–63].

Also, for face-to-face intubation video laryngoscopes are advantageous when compared to standard Macintosh laryngoscopes. VL use for the face-to-face intubation technique is associated with improved visualization, higher first-pass success rates, reduced esophageal intubations, ease of use for less experienced providers, and less physical strain on the operator [49, 51, 64].

Channeled video laryngoscopes, such as the Pentax Airway Scope®, Airtraq® or Kingvision®, which feature a preloaded endotracheal tube, offer benefits such as that they allow the operator to position the laryngoscope in the pharynx with one hand, while the other hand advances the tracheal tube directly into the trachea, guided by the device’s channel. This method requires fewer motor skills than traditional TI, potentially simplifying the process for both novice and experienced practitioners [35, 36, 52, 62, 65].

#### Digital intubation

Digital intubation involves placing an endotracheal tube into the trachea by guiding it with the provider’s fingers without direct visualization. The provider inserts his fingers into the patient’s mouth to locate the epiglottis and the vocal cords through tactile feedback. Once the vocal cords are identified, the endotracheal tube is advanced blindly through the cords into the trachea. With very limited clinical data, digital intubation has been described as a technique used in confined spaces airway management [3–5, 24, 40, 50, 66].

Despite its potential advantages, digital intubation comes with significant challenges. The success relies heavily on the provider’s experience, anatomical knowledge, and tactile skills, as there is no visual confirmation. Studies indicate that digital intubation generally has a lower success rate and may take longer compared to other intubation methods. Additionally, the lack of direct visualization increases the risk of complications, such as misplacement of the tube or trauma to the airway. Furthermore, digital intubation is described as unsafe and uncomfortable for the rescuer [40, 50].

#### Emergency front-of-neck access (eFONA)

Emergency Front-of-neck access (eFONA) may be advantageous in confined spaces [32, 33, 67]. Cricothyrotomy has demonstrated a relatively high success rate compared to TI and SGA in confined spaces. One prospective randomized manikin study found a failure rate of only 2% for open surgical airway techniques compared to a significantly higher failure rate of 65% for needle cannula techniques highlighting the surgical cricothyrotomy over other methods [68]. The success rates of surgical airway techniques in confined spaces appear to be significantly influenced by the level of training and experience of the personnel involved. Emergency physicians were found to have higher success rates in performing cricothyrotomy compared to less qualified providers [67, 69]. For entrapped patients requiring immediate airway management—particularly those with severe maxillofacial trauma or burns—eFONA may be the most appropriate first-line approach, especially if significant delays or challenges with SGA or tracheal intubation are anticipated.

### Rescuer position

During TI of supine patients on the ground, various rescuer positions relative to the patient have been proposed, allowing adaptation to the patient’s specific needs and the constraints of the surrounding environment. Nonetheless, it is important to be aware that any of these positions may be associated with the worse laryngoscopic view and ease of TI compared to an optimal patient positioning on a raised stretcher [70].

**Fig. 1:**
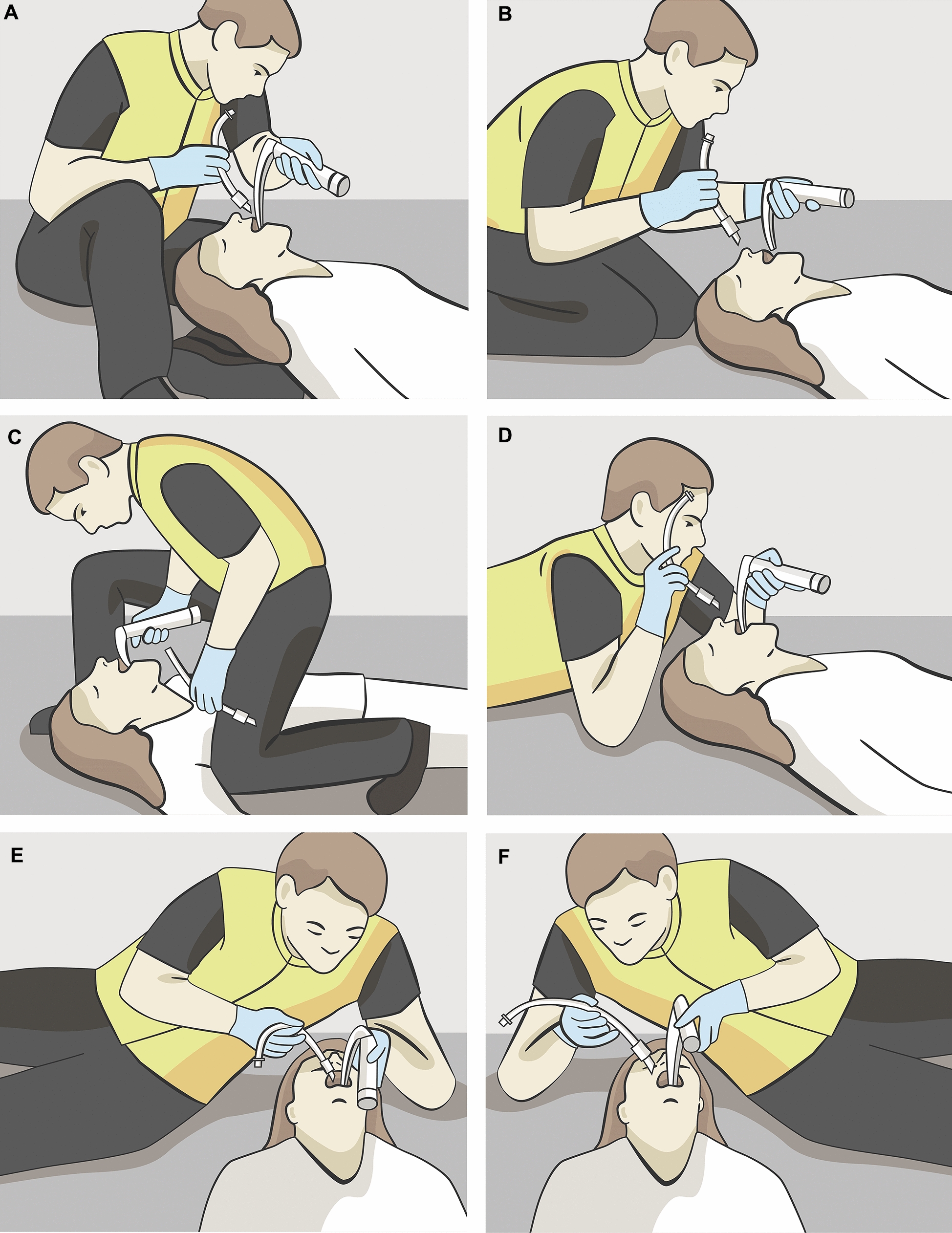
Illustrations of rescuer positioning for tracheal intubation. **A** Sitting position, **B** kneeling position, **C** straddling position, **D** prone position, **E** left lateral position, **F** right lateral position

In the sitting position the rescuer is sitting with patient’s head resting on his bent left leg, while his right leg is extended (Fig. 1A). This allows for good visibility, and rescuers can maintain a comfortable posture, which may reduce fatigue during the procedure. It can also facilitate better communication with other team members. However, it may not provide optimal access to the airway, potentially making it more difficult to achieve proper alignment of the larynx for intubation [71].

Kneeling at the patient’s head provides a stable platform and allows for good visualization of the larynx (Fig. 1B). Kneeling however, may be uncomfortable for the rescuer over extended periods, and it might limit the rescuer’s ability to exert maximum force for alignment of the airway tissues, especially in patients with anatomical difficult airways. The kneeling position does not allow proper alignment of the glottic axis since the operator’s head must be very low for alignment to occur [71, 72].

In the straddling position, the rescuer places their right knee between the patient’s left arm and torso, while their left foot is positioned above the patient’s right shoulder near the head (Fig. 1C). The laryngoscope is held in the rescuer’s right hand. The straddling position allows for a strong body position and enables the rescuer to exert greater force, which can help in aligning the airway more effectively. This position also allows for easier manipulation of the laryngoscope and tracheal tube. In addition, the straddling position has further advantages in that it may provide front access to patients who are trapped in confined spaces. Yet, the straddling position is the most awkward and uncomfortable for the rescuer, and it must be combined with the face-to-face/inverse laryngoscopy mentioned above. Additionally, it might not be feasible with larger patients or with on-going chest compressions [71, 72].

Another option is intubating in the prone position, where the rescuer is lying on the ground, propped up on their elbows behind the patient’s head (Fig. 1D). The prone position may allow for better alignment of the airway, potentially improving visualization of the larynx. However, the prone position can be challenging for shorter rescuers, who may struggle to stabilize their elbows on the ground, impacting their ability to perform the procedure effectively. The prone position necessitates about a meter of extra space at the patient’s head compared to other positions like kneeling or sitting, potentially limiting its applicability in confined spaces. Additionally, it can limit the rescuer’s ability to apply force effectively and may lead to delays in intubation [71–74].

The left and right lateral positions of the rescuer offer several advantages (Fig. 1E and F). In the left lateral position, the left forearm acts as a lever during exposure, minimizing effort during the exposure whereas the right arm remains free to permit tube placement and suctioning. This may be an advantage for the right-handed rescuer. Furthermore, it reassembles the standard left-handed intubation technique. The left lateral position has been associated with a lower incidence of laryngoscopic difficulty compared to other positions. One study found that laryngoscopic difficulty was significantly lower in the left lateral group (11.1%) compared to the kneeling group (26.9%) [71–74].

In the prone, right, and left lateral positions, the rescuer’s head is lower, thus bringing the visual axis in line with the glottic axis.

### Patient position

Tracheal intubation may be conducted in a lateral position to allow continuous airway toilet during TI. Various publications describe the advantages of TI in the lateral position using DL, Intubating LMAs (ILMA), and the Pentax-AWS Airway Scope. While evidence is sparse, lateral positions may help maintain an open airway and reduce the risk of aspiration in unresponsive patients. Laryngoscopic views may be less favorable in lateral positions, however several studies have found similar intubation times when compared to supine patients [52, 75–79].

The lateral position may be particularly helpful when managing patients with severe facial injuries with severe ongoing hemorrhage. Likewise, the TI in the prone position may be an option in specific scenarios [80].

### In cabin/in flight intubation

The difference between **in-cabin intubation** and **in-flight intubation** primarily lies in the environment, challenges, and circumstances surrounding the procedure. **In-Cabin intubation** is performed while the aircraft is on the ground (e.g., in a cabin or a medical transport vehicle) while **in-flight intubation** is performed while the aircraft is in the air (e.g., in an air ambulance or during a commercial in-flight emergency). In-cabin intubation allow for better equipment setup and preparation. In contrast, in-flight intubation is more challenging and unpredictable due to turbulence, noise, vibrations, reduced light, space and altitude changes. Furthermore, there may be limited access to stowed away equipment or by factors not inherent to flight such as reduced workspace, poor positioning, and suboptimal cabin configuration.

The existing literature on in-cabin or in-flight TI is limited and difficult to compare due to significant variability in clinicians’ backgrounds, standards of practice, and training, which may not be directly comparable. Furthermore, these studies involve differing helicopter models and cabin configurations [47, 81–91].

Several studies have demonstrated success rates for in-cabin intubation comparable to intubation achieved in preflight or hospital settings [81–83, 85–90] while one other study found significantly lower success rates [85, 91]. A systematic review analyzed studies incorporated data from three randomized controlled trials that assessed first-pass intubation success, intubation time, and difficulty of intubation. The authors concluded that protocolized in-cabin intubation can be executed successfully, with less perceived difficulty, and in a timely manner, with conditions equal to or better than those found in outdoor settings. Yet, there was a slight delay in securing the airway, the overall reduction in scene time may beneficial [86].

A protocolized setup specific to the aircraft and interior design provides a clear and systematic method for in cabin intubation [47, 82, 83].

## Conclusion

Prehospital providers frequently encounter patients in confined spaces, both in controlled and uncontrolled environments. Based on our group’s experience, proper planning and training both on an institutional and an individual level are essential to mitigate complications. Protocolization and planning confer numerous benefits when working in controlled environments, these benefits likely transfer to uncontrolled environments as well.

Managing prehospital patients in confined spaces requires integration of medical care and logistical planning, while keeping momentum toward extrication as one of the primary goals. Airway management inherently slows or interrupts these efforts. Therefore, traditional indications for definitive airway management may not apply to these scenarios. Whenever possible, airway management should prioritize maintaining an open airway and oxygenation rather than achieving a definitive airway post extrication.

If airway management is required several points should be considered. Bag mask ventilation should only be used as a temporary bridge to more reliable airway strategies. Supraglottic devices appear to offer advantages in confined spaces and should be considered a first line approach. TI should be deferred until the patient has been extricated if possible. Emergency Front of Neck Access using a surgical technique has shown the highest success rate in all manikin studies we reviewed.

Tracheal intubation with the patient or provider in an atypical position is a high-risk procedure and should be considered a last resort, as it requires modification of technique or the use of adjuncts, different devices and is associated with increased risk of esophageal tube placement.

## Data Availability

No datasets were generated or analysed during the current study.
